# Histologic and Histomorphometric Comparison between Sintered Nanohydroxyapatite and Anorganic Bovine Xenograft in Maxillary Sinus Grafting: A Split-Mouth Randomized Controlled Clinical Trial

**DOI:** 10.1155/2017/9489825

**Published:** 2017-08-06

**Authors:** Claudio Stacchi, Teresa Lombardi, Francesco Oreglia, Andrea Alberghini Maltoni, Tonino Traini

**Affiliations:** ^1^Department of Medical, Surgical and Health Sciences, University of Trieste, Trieste, Italy; ^2^Private Practice, Cassano allo Ionio, Italy; ^3^Private Practice, Verona, Italy; ^4^Private Practice, Firenze, Italy; ^5^Department of Medical, Oral and Biotechnological Sciences, University of Chieti-Pescara, Chieti, Italy

## Abstract

The presence of vital bone after maxillary sinus augmentation is crucial to enhance the quality of bone-implant interface, ensuring predictable long-term results. The aims of this RCT with split-mouth design were the histologic and histomorphometric comparison of two different biomaterials in sinus elevation after 6 months of healing and the evaluation of the clinical outcomes of implants inserted in the augmented areas after 12 months of prosthetic loading. Twenty-eight patients (10 females, 18 males) were treated with bilateral sinus floor elevation with lateral approach. Pure sintered nanohydroxyapatite (NHA) and anorganic bovine bone (ABB) were used as test and active control, respectively. After six months, 52 bone biopsies were harvested from 26 patients, and 107 implants were inserted in the augmented areas. Histomorphometry showed that, in the two groups, vital bone percentages were 34.9 ± 15% (NHA) and 38.5 ± 17% (ABB) (*p* = 0.428), marrow spaces percentages were 44.5 ± 18% (NHA) and 43.5 ± 23% (ABB) (*p* = 0.866), and residual graft percentages were 20.6 ± 13% (NHA) and 22.3 ± 12% (ABB) (*p* = 0.638). After 6 months of healing, no statistically significant difference was present in histomorphometric outcomes between NHA and ABB groups. Implant survival rate in NHA group after 12 months of loading was 96.4%, showing no statistically significant differences with ABB group.

## 1. Introduction

Bone resorption and sinus pneumatization are common occurrences in the posterior maxilla after tooth extraction: they may cause both a quantitative reduction and qualitative deterioration of bone, resulting in an inadequate bone volume for dental implant placement [1]. Sinus floor elevation technique had been described more than 35 years ago [2] and extensively studied afterwards, demonstrating high predictability in regenerating bone and allowing for reliable implant supported rehabilitation [3, 4]. Accurate presurgical planning is a mandatory step: sinus three-dimensional conformation, eventual presence of Underwood septa, and precise localization of the alveolar-antral artery should be assessed and carefully evaluated in order to minimize intraoperative complications and optimize surgical techniques [5–7].

In general, the quality of osseointegration of dental implants is directly related to the bone-implant contact: in a regenerated tissue, the quantity of newly formed bone is of paramount importance for successful integration of the fixture. Therefore, an adequate biomaterial choice is the first crucial step in bone-implant interface engineering to ensure positive clinical long-term results.

Autogenous bone had been the first grafting material to be used in sinus floor elevation, being considered as the gold standard option for a long time [8], but its tendency of resorption, its limited availability, and the necessity of a donor site with associated increased morbidity for the patient should be considered as major drawbacks. In the attempt to overcome these limitations, many biomaterials had been proposed and tested, such as allografts, *β*-tricalcium phosphate, calcium sulphate, and bone mineral matrix [9–15]. However, anorganic bovine bone (ABB) is probably the most widely investigated bone substitute and, when used in sinus floor elevation, demonstrated satisfactory osteoconductive properties and dimensional stability: from a clinical point of view, implants inserted in ABB-grafted areas showed a high survival rate over time [16, 17].

Also, synthetic hydroxyapatites, when used as a bone graft, showed a high degree of biocompatibility and support for cellular activity: they stimulate osteoconduction and are generally slowly replaced by the host bone after implantation [18]. Continuous improvements in synthesis, manufacturing technology, and purification have led to a variety of synthetic HA-based materials with different properties. Among them, synthetic hydroxyapatite with nanoscale porosities seems to favor bone matrix proteins adhesion and to promote differentiation of osteoblast precursor cells [19, 20]. Even if nanocrystalline HA embedded in a highly porous silica gel matrix was already tested as a material for sinus floor elevation [21, 22], a prospective study with a direct comparison between the behavior in the maxillary sinus of pure sintered nanohydroxyapatite (NHA) and ABB in terms of osteoconductive potential was not performed yet.

The aim of this parallel-group, superiority randomized clinical trial (RCT) with split-mouth design was the histologic and histomorphometric comparison for the newly formed tissue after sinus floor elevation with lateral approach performed by using two different grafting materials: NHA as test and ABB as active control.

An additional aim was the evaluation of the clinical outcomes of dental implants inserted in the augmented areas after 12 months of prosthetic loading. The planned follow-up for this study is five years after prosthetic loading.

## 2. Materials and Methods

### 2.1. Study Design

The present study was a multicenter randomized controlled clinical trial with a split-mouth design, following CONSORT guidelines, and was conducted in four clinical centers in accordance with the Good Clinical Practice Guidelines (GCPs) and with the recommendations of the Declaration of Helsinki as revised in Fortaleza (2013) for investigations with human subjects. The study protocol had been approved by the relevant ethical committee (Comitato Etico Calabria-Sezione Area Nord) and registered in a public register (NCT03077867).

Patients were thoroughly informed about the protocol, the treatment and its alternatives, the benefits, and the possible risks and signed written informed consent for the participation in the study. This superiority trial tested the null hypothesis of no differences in new bone formation and dental implant survival between NHA bone grafts (test group) and ABB grafts (active control group) in atrophic maxillae treated with sinus floor elevation with lateral approach.

### 2.2. Study Population

Eligible participants were adult patients (aged ≥ 18 years), with severe bilateral maxillary atrophy (crestal height < 3 mm, class V-VI of Cawood and Howell classification [23]) and needing sinus floor elevation to allow for fixed rehabilitation supported by osseointegrated implants, inserted with a staged approach.

Exclusion criteria were

1. acute myocardial infarction within the past 2 months;
2. uncontrolled coagulation disorders;
3. uncontrolled diabetes (HbA1c > 7.5%);
4. radiotherapy to the head/neck district within the past 24 months;
5. immunocompromised patients (HIV infection or chemotherapy within the past 5 years);
6. present or past treatment with intravenous bisphosphonates;
7. allergy to bovine collagen;
8. presence of uncontrolled or untreated periodontal disease;
9. presence of sinusal pathologies contraindicating sinus floor elevation procedures;
10. psychological or psychiatric problems;
11. alcohol or drugs abuse;
12. patient not fully able to comply with the study protocol;
13. Schneiderian membrane perforation during surgery.

### 2.3. Surgical Procedures

Surgical procedures were performed in four centers by experienced operators (CS, TL, FO, and AAM). Patients were draped to guarantee maximum asepsis and perioral skin was disinfected by using iodopovidone 10% (Betadine, Medifarm, Italy). After performing local anesthesia by using articaine 4% with epinephrine 1 : 100.000 (Artin, Omnia, Italy) and raising a full-thickness flap, a window was designed on the lateral wall of the sinus by using ultrasonic instrumentation with the erosion technique (Piezosurgery Touch, Mectron, Italy, and Piezotome, Acteon, France) [24] and the Schneiderian membrane was carefully elevated using ultrasonic inserts and manual curettes. After checking the integrity of the Schneiderian membrane with Valsalva maneuver, the randomization sealed opaque envelope was opened, revealing to the surgeon the grafting material to be used. The biomaterials selected for this study were sintered NHA (Fisiograft Bone Granular, Ghimas, Italy) in the test sites and ABB (Bio-Oss, Geistlich, Switzerland) in the control sites. After the completion of the grafting procedure, the antrostomy was covered by a resorbable bovine collagen membrane (Bio-Gide, Geistlich, Switzerland), fixed with two pins (Micropin, Omnia, Italy), and flaps were sutured with Sentineri technique [25] and single stitches using a synthetic monofilament (PTFE, Omnia, Italy).

The contralateral sinus floor augmentation was performed in the same surgical session, with the same surgical protocol, inserting the grafting material not selected in the first intervention.

Patients were prescribed antibiotics for 6 days (amoxicillin 1 g twice a day or, in allergic patients, clarithromycin 250 mg three times a day) and NSAID (ibuprofen 600 mg), when needed.

All patients were also advised to sneeze with the mouth open and to avoid nose blowing for two weeks, to prevent unnecessary pressure on the sinus membrane.

Sutures were removed 10 days after surgery. Postsurgical visits were scheduled at monthly intervals to check the course of healing. After six months, bone-core biopsies were collected from the grafted areas using a trephine bur (3.5 mm diameter) during the implant bed preparation, and then dental implants (BnxEvo, Ghimas, Italy) were inserted in the harvesting sites. Bone-core specimens were collected with the assistance of surgical templates based upon individual prosthetic requirements. The surgical guides were also used to insert the other programmed implants into the augmented areas: they were left submerged for a four-month healing period, prior to being connected to healing abutments. Finally, implants were restored with screwed metal-ceramic prostheses and patients were followed up for twelve months after loading.

### 2.4. Histological Analysis

Bone biopsies, left inside the trephine burs, were carefully rinsed for 30–40 seconds with a cold 5% glucose solution to remove blood maintaining the correct osmolarity (278 mOsm/L).

The specimens were then placed in Eppendorf tubes with an adequate volume (at least ten times the volume of the specimen) of 10% formalin solution buffered with phosphate to pH 7.2.

Each specimen was stored in a separate container and labeled. Both patient name and operator ID were noted on a separate sheet to identify the specimens. During the processing phase, both patient name and operator ID were designated by a numerical code.

The specimens were rinsed twice with phosphate-buffered saline and dehydrated with a graded series of alcohol at 4°C for seven days. Complete dehydration was then obtained with absolute alcohol immersion for two additional days. Subsequently, the specimens were preinfiltrated in a 50% resin/alcohol solution (Technovit 7200 VLC, Kulzer, Germany) for ten days and completely embedded in 100% resin (two changes) using a vacuum chamber for twenty additional days or until the specimens have become transparent.

Finally, specimens were easily removed from the trephine bur using a custom-made plunger (thanks to the shrinkage consequent to dehydration and resin infiltration) and then oriented and polymerized.

After polymerization, the specimens were cut along the longitudinal axis using a high-precision diamond disc at about 50 microns (TT System, TMA2, Italy). The sections were ground under running water to about 30 ± 10 microns using a series of polishing discs from 400 to 1200 grits, followed by a final polish with 0.3-micron alumina in a microgrinding system (TT System, TMA2, Italy).

The prepared sections were stained with Toluidine Blue and Azure II and counterstained with acid fuchsin or double-stained with Toluidine Blue with Pironine G at 1% and Azure II. The investigation was conducted in a transmitted brightfield microscope (BX 51, Olympus America, USA) and under brightfield/circularly polarized light microscope (Axiolab, Zeiss, Germany) both connected to high-resolution digital cameras (FinePix S2 Pro, Fuji Photo Film, Japan).

Digital photomicrographs were used for histomorphometric analysis, which was performed by a trained and experienced operator (TT). The following parameters were measured: (1) amount of tissue collected with the biopsies over the obtained sections (size of samples); (2) amount of vital bone as absolute value (mm^2^) and as relative value (vital bone area/total sample size × 100); (3) marrow space (connective tissue) as absolute value (mm^2^) and as relative value (connective tissue area/total sample size × 100); (4) residual grafting material as absolute value (mm^2^) and as relative value (biomaterial area/total sample size × 100). A histometric software package with image capturing capabilities (Image-Pro Plus 6.0, Media Cybernetics Inc., USA) was used. To ensure accuracy, the software was calibrated for each experimental image using a feature named “Calibration Wizard,” which creates a linear remapping of the pixel numbers in millimeters. Intraexaminer variability was controlled by carrying out two measurements for each controlled index. When the difference between the two performed readings exceeded 5% for the same index, the measurement was repeated.

### 2.5. Outcomes

This study evaluated the following outcome measures:

- Quality of the newly formed tissue: (1) new bone formation (percentage of newly formed bone area to total measured area), (2) residual graft particles (percentage of graft particles area to total measured area), and (3) marrow spaces (percentage of soft-tissue area to total measured area).
- Implant failure: implant mobility and/or any situation suggesting implant removal.
- Biological and mechanical complications: any complication defined as an unexpected deviation from the normal treatment outcome, both biological (membrane perforation, hemorrhagic events, sinusitis, peri-implantitis, etc.) and mechanical (implant fracture, prosthesis fracture, fixation screw loosening, etc.)

### 2.6. Sample Size and Statistical Power

Maxillary sinuses were divided into two groups, depending on the grafting material used: a sample size of 24 sinuses per group was needed to detect an effect size of 0.6 on the quantity of newly formed bone (primary outcome), referred to as indicative of a medium effect [26], between the groups (alpha level set at 0.05 and power of 80%) (DSS Research, Fort Worth, USA). The effect size is defined as the difference in the given outcome between the groups divided by the within-subjects standard deviations. Each clinical center treated 7 patients with bilateral sinus floor elevation for a total of 56 augmented sinuses to compensate eventual dropouts occurring during the study.

### 2.7. Randomization

A computer-generated table, distributing right and left sinuses of each patient into two groups (test and control), was prepared using a balanced, randomly permuted block approach (http://www.randomization.com/). The randomization codes were enclosed in numbered, sealed, opaque envelopes which were opened after Schneiderian membrane elevation. Therefore, treatment allocation was concealed to the surgeons in charge of recruiting and treating the patients included in this clinical trial.

### 2.8. Statistical Analysis

Statistical analysis was performed by means of a computerized statistical package (SigmaStat 3.5, SPSS Inc., Germany). Data were expressed as mean ± SD and median (interquartile range), respectively, for parametric and nonparametric values. Items were analyzed with descriptive statistics to assess whether they had a normal distribution; both equal variance and normality tests were used. Considering the two-arm superiority RCT study design, the hypothesis was tested using unpaired *t*-test in case of normally distributed data, while Mann–Whitney  *U* test was performed to compare nonparametric values. A *p* value < 0.05 was considered statistically significant.

## 3. Results

### 3.1. Clinical Results

Twenty-eight patients (aged 60.1 ± 10.7 years, range: 39–79 years, 10 females, 18 males) were enrolled, randomized, and treated with bilateral sinus floor elevation with lateral approach. Each clinical center contributed with 7 patients. Eighteen patients were nonsmokers, six light smokers, and four heavy smokers. Preoperative residual bone crest height ranged from 0.5 to 3 mm (mean height: 2.03 ± 0.75 mm). The main baseline patient characteristics are summarized in Table 1.

Three sinus membrane perforations occurred during elevation in two patients, who dropped out from the study. However, membrane perforations were covered by multiple layers of A-PRF membranes (PRF Duo, Mectron, Italy) and grafting procedures were successfully completed in all of the three cases. No other intraoperative complications were recorded. The healing period following sinus augmentation was uneventful in all patients.

Six months after sinus floor elevation procedures, 52 bone biopsies were harvested from 26 patients (26 biopsies in test sites, 26 in control sites), and a total of 107 implants were inserted and submerged under the soft tissues (55 implants in test sites, 52 in control sites). After four additional months, at healing abutments connection, three implants in three patients resulted to be not osseointegrated (2.8% cumulative failure rate): two implants were inserted in test sites (3.6% failure) and one implant was inserted in a control site (1.9% failure). Difference in implant failure rate between test and control groups was not statistically significant (*p* = 0.32, unpaired *t*-test). After an accurate debridement of the implant bed, removed fixtures were immediately replaced with larger diameter implants, which were restored after four additional months of healing. Metal-ceramic screwed prostheses were delivered and, at 12-month follow-up, all implants and prostheses were successfully in function without the occurrence of any biological or mechanical complication.

### 3.2. Histomorphometric Results

**Figure 1:**
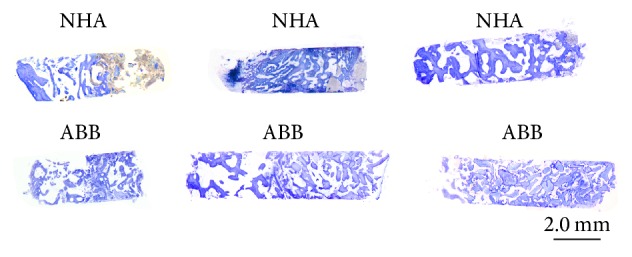
Sections of bone cores retrieved after 6 months of healing. NHA: sintered nanohydroxyapatite; ABB: anorganic bovine bone (Toluidine Blue and Azure II; original magnification: 12x).

The sections of the harvested biopsies had a mean surface of 9.05 ± 2.7 mm^2^ for NHA group and 10.31 ± 2.9 mm^2^ for ABB group. The difference between the two groups was not statistically significant (*p* = 0.116, unpaired *t*-test). Area of vital bone was 3.29 ± 2.1 mm^2^ for NHA group and 4.12 ± 2.9 mm^2^ for ABB group. The difference between the two groups was not statistically significant (*p* = 0.213, Mann–Whitney *U* test). Connective tissue area was 3.82 ± 1.5 mm^2^ for NHA group and 4.09 ± 2.3 mm^2^ for ABB group. The difference between the two groups was not statistically significant (*p* = 0.869, Mann–Whitney *U* test). The area occupied by residual grafting material was 1.92 ± 1.4 mm^2^ for NHA group and 2.09 ± 1.4 mm^2^ for ABB group. The difference between the two groups was not statistically significant (*p* = 0.516, Mann–Whitney *U* test). The results are summarized in Table 2 and Figures 1() and 2().

**Figure 3:**
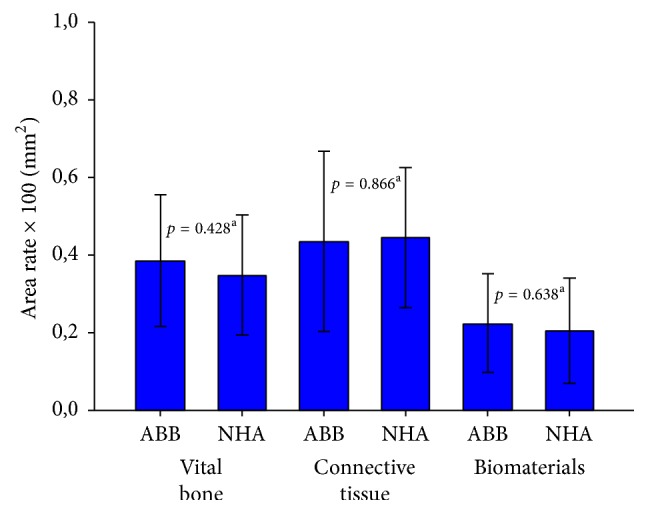
Histomorphometric measurements expressed in percentage. NHA: sintered nanohydroxyapatite; ABB: anorganic bovine bone. (a) Unpaired *t*-test. Level of significance: *p* < 0.05.

The average percentage of vital bone was 34.9 ± 15% for NHA group and 38.5 ± 17% for ABB group. The difference between the two groups was not statistically significant (*p* = 0.428, unpaired *t*-test). The average percentage of connective tissue was 44.5 ± 18% for NHA group and 43.5 ± 23% for ABB group. The difference between the two groups was not statistically significant (*p* = 0.866, unpaired *t*-test). The average percentage of residual grafting material was 20.6 ± 13% for NHA group and 22.3 ± 12% for ABB group. The difference between the two groups was not statistically significant (*p* = 0.638, unpaired *t*-test). Results are summarized in Table 3 and Figure 3().

## 4. Discussion

In the clinical practice, the final purpose of bone regeneration is the formation of an adequate quantity of tissue of good quality, in which to insert dental implants with a good long-term prognosis. Obviously, the biological behavior of the biomaterials is of primary interest. Kirkpatrick et al. underlined the differences between the regenerative processes that have the teleological purpose of “restitutio ad integrum” of the affected tissue and the repair process, which is a structural adaptation to a function task [27]. It was reported that the bone regeneration process with alloplastic, xenograft, and allograft bone substitutes follows three main phases: T1 which is the “time of grafting,” with a predominant heterogeneous phase in suspension of blood clot and particles of biomaterials; T2 which is the “time of repairing,” with a solid heterogeneous composite phase of biomaterial remnants and newly formed bone; T3 which is the “time of regeneration,” with a solid homogeneous phase of newly formed bone without biomaterials remnants. The most common bone substitute biomaterials do not reach the phase T3 in their clinical use [28]. However, many studies demonstrated that implant osseointegration process can be also obtained and maintained in augmented sinuses where residual graft particles were still present, without a negative influence of biomaterial remnants on peri-implant bone regeneration [29–33].

Hence, even if autologous bone had been traditionally considered as the gold standard to promote new bone regeneration, the choice of alternative biomaterials is now the preferred option in sinus floor elevation for three main reasons: less morbidity, less resorption, and unlimited availability. ABB is the most widespread biomaterial used for sinus grafting and its behavior had been extensively investigated over the years, showing satisfactory long-term results [16, 17]. However, disadvantages of xenografts should also be considered: they include potential risk of prion disease transmission [34] and reaction of the host immune system [35]; in addition, some patients could refuse their use for religious motivations or because they are in contrast with their lifestyle (e.g., vegans and vegetarians). In a recent study, allografts and xenografts elicited the highest refusal rates among the surveyed patients: 15% of the patients said they would accept a xenograft under no circumstances, while 18% said they would accept this type of bone graft only as a last resort [36].

The use of synthetic, alloplastic biomaterials could overcome these limitations: they have been studied for years and successfully used in sinus augmentation, but a direct comparison with xenografts, in a split-mouth design, has been reported in the literature by very few and often underpowered trials [15, 37–39].

The results of the present RCT showed no statistically significant differences between NHA and ABB groups in terms of new bone formation and survival rate of implants inserted in the augmented areas after 12 months of prosthetic loading: therefore, the null hypothesis tested in this study was accepted.

The histometric comparison after 6 months of healing showed that the osteoconductive potential of NHA is clinically and statistically comparable to ABB, even if it resulted in a slightly lower percentage of vital bone (34.9% against 38.5%), but showing also a lower percentage of residual grafting material (20.6% against 22.3%). Our data are in accordance with other human studies performed by using synthetic hydroxyapatites as sinus grafting material, reporting new bone formation at six months ranging from 32 to 38.5% [40–42]. These results are also comparable with the histomorphometric outcomes of sinus augmentation performed by using solely autogenous bone (new bone formation at six months ranging from 36.8 to 41%) [41, 43–45].

**Figure 4:**
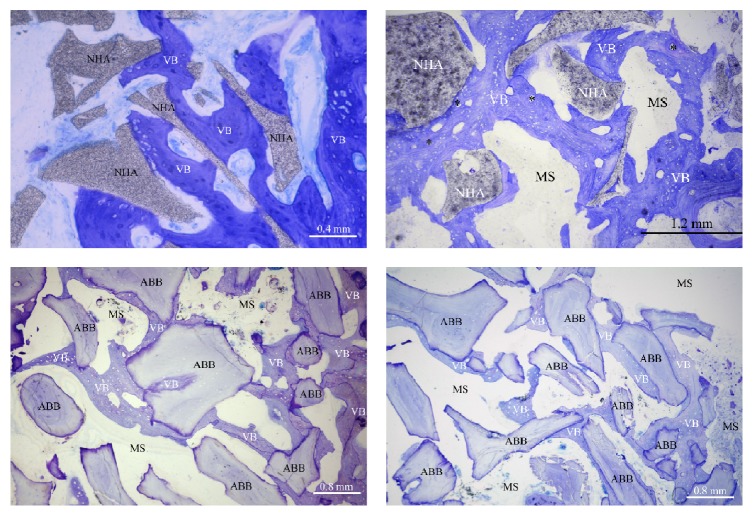
Biomaterial particles of both groups appeared to be surrounded and merged by newly formed bone. Several haversian systems (*∗*) were noted in the newly formed bone. NHA: sintered nanohydroxyapatite; ABB: anorganic bovine bone; VB: vital bone; MS: marrow spaces (Toluidine Blue and Azure II; original magnification: 100x).

**Figure 5:**
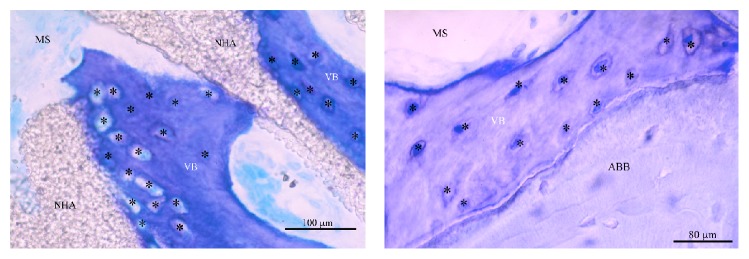
The bone around both biomaterials was characterized by the presence of osteocytes (*∗*) embedded inside the mineralized bone matrix. In the test group, osteocytes were generally more numerous near the material surface. NHA: sintered nanohydroxyapatite; ABB: anorganic bovine bone; VB: vital bone; MS: marrow spaces (Toluidine Blue and Azure II; original magnification: 400x).

**Figure 6:**
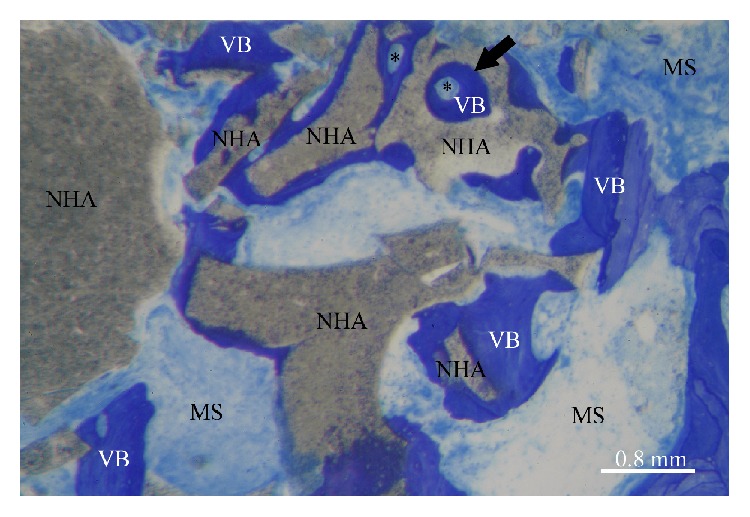
The new bone around some NHA particles presented osteons (*∗*), indicating a relative angiogenetic potential of the material. Vessels' growth was present in pores of adequate dimension inside the biomaterial (black arrow). NHA: sintered nanohydroxyapatite; ABB: anorganic bovine bone; VB: vital bone; MS: marrow spaces (Toluidine Blue and Azure II; original magnification: 100x).

**Figure 7:**
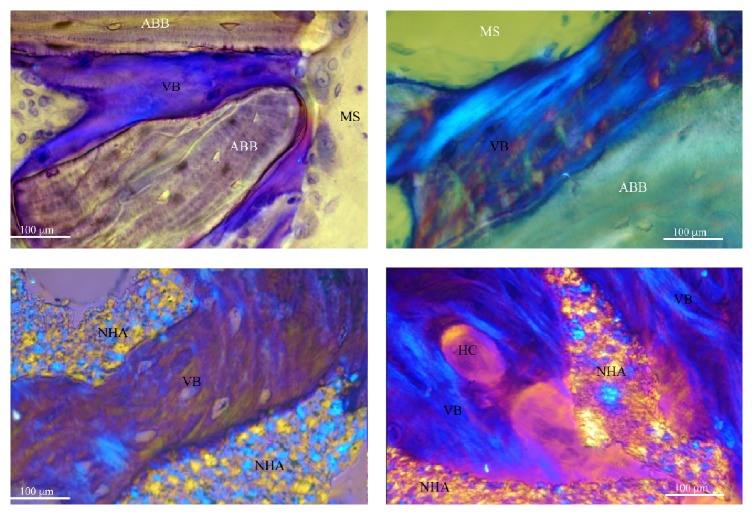
Under circularly polarized light, osteoconduction appeared clear with intimate contact between the new bone (VB) and both biomaterials (NHA and ABB). Moreover, the differences in the microstructure (mainly collagen fiber orientation) can be observed. Around some NHA particles, the new bone presented osteons with vessels (HC).

After 6 months of healing, both biomaterials used in the present study showed a good level of “osseointegration,” with an adequate extension of bonding surface between host bone and the biomaterial particles. Graft remnants were easily recognizable from the other components of the regenerated tissue and appeared to be merged by bridges of new bone (Figure 4()). Furthermore, the bone around the biomaterial particles was characterized by numerous osteocytes embedded into the mineralized bone matrix. These cells, in the test group, were generally more numerous near the biomaterial surface (Figure 5()): this feature indicates both a considerable osteointegrative property due to stimulation of the osteoblastic activity and an osteoinductive property of the external surface of the biomaterial (Figure 6()). These aspects, according to other studies [46–48], seem particularly related to NHA structure. As described in detail by Kasai et al. [49], cells' proliferation appeared to be stimulated, when in contact with NHA paste, by the activation of epidermal growth factor receptor (EGFR) and its downstream targets serine/threonine protein kinase (AKT) and signal regulated kinases (ERK 1/2). Finally, as expected, both groups showed intense osteoconductive activities (Figure 7()).

Implant survival rate in NHA group after 12 months of loading (96.4%) showed no statistically significant differences with ABB group. This outcome is also comparable with results reported in recent systematic reviews for sinus grafting using solely autogenous bone (97.2–97.4%) or different bone substitutes (98.2–98.6%) [50, 51].

Currently, the main limitation of the present study is the relatively short time of follow-up: however, a long-term evaluation of the clinical outcomes in the patients enrolled in this trial had already been coordinated. Furthermore, additional investigations on the biomechanical performances of different bone substitutes would help in determining their appropriate clinical use.

## 5. Conclusions

The findings of the present RCT showed that both NHA and ABB led to the formation of a regenerated tissue composed of more than 1/3 of vital bone after six months of healing, without any statistically significant difference between test and control groups. NHA could be regarded as a suitable grafting material for clinical cases needing bone augmentation to allow dental implant placement.

The clinical implications of the present study include the possibility of increasing the alternatives for the replacement of bone autografts, which not always represent a possible or convenient option. Sintered NHA couples the benefits of technological advancement with the safety of synthetic biomaterials, preventing the potential risks of xenograft implantation to the patient.

## Figures and Tables

**Figure 2:**
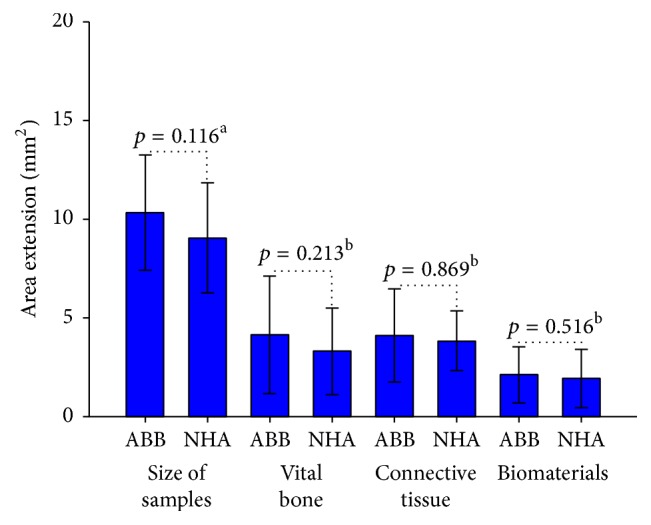
Samples area (mm^2^) and surface of vital bone, connective tissue, and biomaterial remnants (mm^2^). NHA: sintered nanohydroxyapatite; ABB: anorganic bovine bone. (a) Unpaired *t*-test; (b): Mann–Whitney *U* test. Level of significance: *p* < 0.05.

**Table 1:** Patient characteristics at baseline.

Males	18 (64.3%)
Females	10 (35.7%)
Mean age (range)	60.1 (39–79)
Nonsmoker	18 (64.3%)
Light smoker (<10)	6 (21.4%)
Heavy smoker (≥10)	4 (14.3%)
Mean residual bone (SD) (range) (mm)	2.0 (0.7) (0.5–3)

**Table 2:** Total area of the analyzed sections [mm^2^].

	Samples	Missing	Mean	Std. dev.	Std. error	CI of mean
Total area ABB	26	0	10,319	2,923	0,573	1,180
Total area NHA	26	0	9,052	2,796	0,548	1,129
Vital bone ABB	26	0	4,129	2,992	0,587	1,208
Vital bone NHA	26	0	3,297	2,199	0,431	0,888
Connective ABB	26	0	4,090	2,355	0,462	0,951
Connective NHA	26	0	3,828	1,510	0,296	0,610
Biomaterial ABB	26	0	2,099	1,421	0,279	0,574
Biomaterial NHA	26	0	1,926	1,486	0,291	0,600

	Range	Max.	Min.	Median	25%	75%

Total area ABB	12,190	18,340	6,150	9,920	8,720	11,120
Total area NHA	10,600	14,480	3,880	8,550	7,210	11,000
Vital bone ABB	15,820	16,950	1,130	3,705	2,530	5,040
Vital bone NHA	9,700	9,960	0,260	2,750	1,840	4,630
Connective ABB	7,920	8,470	0,550	3,815	1,920	6,230
Connective NHA	6,320	6,920	0,600	3,900	2,960	4,940
Biomaterial ABB	5,480	5,540	0,0600	1,795	1,200	3,110
Biomaterial NHA	5,380	5,530	0,150	1,530	0,810	2,770

	Skewness	Kurtosis	K-S dist.	K-S prob.	Sum	Sum of squares

Total area ABB	0,916	0,972	0,161	0,080	268,290	2981,976
Total area NHA	0,260	−0,524	0,111	0,515	235,340	2325,652
Vital bone ABB	3,279	13,938	0,221	0,002	107,360	667,054
Vital bone NHA	1,232	2,081	0,123	0,380	85,730	403,549
Connective ABB	0,139	−1,039	0,107	0,573	106,350	573,633
Connective NHA	−0,240	−0,184	0,137	0,228	99,530	437,977
Biomaterial ABB	0,596	−0,208	0,138	0,222	54,570	165,038
Biomaterial NHA	1,117	0,622	0,168	0,057	50,080	151,657

**Table 3:** Histomorphometric data expressed in percentage [%].

	Samples	Missing	Mean	Std. dev.	Std. error	CI of mean
Vital bone rate ABB	26	0	0,385	0,170	0,0333	0,0685
Vital bone rate NHA	26	0	0,349	0,155	0,0303	0,0625
Connective rate ABB	26	0	0,435	0,232	0,0456	0,0939
Connective rate NHA	26	0	0,445	0,181	0,0354	0,0729
Biomaterial rate ABB	26	0	0,223	0,128	0,0252	0,0518
Biomaterial rate NHA	26	0	0,206	0,135	0,0265	0,0546

	Range	Max.	Min.	Median	25%	75%

Vital bone rate ABB	0,810	0,924	0,114	0,379	0,263	0,469
Vital bone rate NHA	0,661	0,688	0,0270	0,339	0,279	0,465
Connective rate ABB	0,942	0,990	0,0480	0,462	0,293	0,560
Connective rate NHA	0,709	0,786	0,0770	0,453	0,308	0,562
Biomaterial rate ABB	0,442	0,448	0,00600	0,228	0,138	0,310
Biomaterial rate NHA	0,524	0,550	0,0260	0,161	0,130	0,309

	Skewness	Kurtosis	K-S dist.	K-S prob.	Sum	Sum of squares

Vital bone rate ABB	1,256	2,846	0,131	0,288	10,015	4,576
Vital bone rate NHA	−0,143	−0,0594	0,126	0,345	9,079	3,768
Connective rate ABB	0,381	0,0977	0,0961	0,698	11,316	6,276
Connective rate NHA	−0,132	−0,392	0,102	0,625	11,570	5,964
Biomaterial rate ABB	0,0123	−0,727	0,0758	0,858	5,800	1,706
Biomaterial rate NHA	0,840	0,275	0,152	0,124	5,350	1,558
